# Supplementary material for: A new Lower Triassic ichthyopterygian assemblage from Fossil Hill, Nevada
Source: PeerJ. 2016 Jan 26;4:e1626. doi: 10.7717/peerj.1626 (PMC4741062; doi:10.7717/peerj.1626)
Supplement: Supplemental Information 1 [file peerj-04-1626-s001.docx]

**Conodonts from the lower Member of the Prida Formation, Fossil Hill, Nevada**

Five conodont collections were recovered from the Lower Member of the Prida Formation at Fossil Hill. These largely originated from the interval identified (on Figure 1) as the *Prohungarites gutstadi* beds, an ammonoid reported from horizons H11-12 in the Spathian scheme of Guex et al. (2010). All of the conodont collections are dominated by elements of *Triassospathodus symmetricus* (Orchard), in most cases accompanied by fewer elements of *Neostrachanognathus* sp. The first of these taxa are identified by both the typical P1 elements and by the other elements of the multielement apparatus as illustrated by Orchard (2005); in particular, the distinctive S1 element (Orchard, 2005, fig. 19G, not 19H), named *Cypridodella unialata* by Mosher (1968) and later referred partly to *Ellisonia* sp. (Mosher, 1973, pl. 17, fig, 20), is a good proxy for the species. For *Neostrachanognathus*, the most common elements are those interpreted as S elements by Agematsu et al. (2008, figs. 7, 8), although rare P elements are not clearly conspecific with those illustrated from Oman (op. cit.).

*Triassospathodus symmetricus* is commonly the most abundant conodont species found in latest Spathian faunas. A member of the *T. homeri* group, this species lacks the relatively long posterior process of *T. homeri* (Bender) sensu stricto (and the even longer process of *T. anhuiensis* (Ding), but it has a similar stratigraphic distribution. Representatives of the group are the most common and ubiquitous taxa in late Spathian conodont faunas in Eurasia and North America, often occurring to the exclusion of other taxa. In North America, *T. symmetricus* was originally reported from much of the Spathian (Orchard, 1995), although it is far more common in the higher Spathian *Prohungarites* beds and the *Neopopanoceras haugi* Zone, and in British Columbia where the *Keyserlingites subrobustus* Zone contains a very similar fauna. In the Olenekian-Anisian boundary beds of both the Desli Caira section of Romania (Orchard et al., 2007a) and the Guandao section of China (Orchard et al., 2007b), the *homeri* group also ranges into the basal Anisian. However, there are no Anisian indicators in the present fauna, and the relative abundance of the group is similar to that seen in the deeper Spathian levels of Guandao (Orchard et al., 2007b, fig. 4).

*Neostrachanognathus* is known from neither Delsi Caira nor Guandao, but is commonly encountered in North American faunas dominated by *Triassospathodus* and assignable to the Subcolumbites Zone of Guex et al. (2010), which includes strata formerly characterized as the *Prohungarites* or *Stacheites* beds (author’s collections) in Idaho (Hammond Creek), and Nevada (Coyote Canyon-Humboldt Range, Tobin Range, New Pass). The taxon, which was recorded as *Oncodella* n. sp. A by Orchard (1994), is far less common in the lower part of the overlying Haugi Zone in Coyote Canyon, and absent in the higher parts of that zone. *Neostrachanognathus* from the USA may be identical to elements from the Spathian of Oman (Agemetsu et al., 2008), which are interpreted to lie stratigraphically above *Procarnites* -bearing strata (Orchard, 1995, p. 112). The genus is also known from Spathian strata in Nepal (Hatleberg & Clark, 1984), Pakistan (Sweet, 1970 collections: Nammal2, Landa, unpublished), and Spiti (Bucher collection, unpublished). A further record of the genus may be represented by *Oncodella obuti* Buryi from the Spathian of Primorye, Russia (Buryi, 1979).

In summary, the conodonts associated with the ichthyopterygian jaws suggest a late, but not latest Spathian age. It is likely a correlative of conodont Fauna 3 of Orchard (1995).

**References**

Agematsu, S., Orchard, M. J., and Sashida, K. 2008. Reconstruction of an apparatus of *Neostrachanognathus tahoensis* from Oritate, Japan and species of *Nesotrachanognathus* from Oman. *Palaentology*, 51(5): 1201-1211.

Buryi, G., 1979. Lower Triassic conodonts of the South Primorya. Inst. *Geolojii i Geofiziki, Sibirskoe Otdelenie,* Akad Nauk, SSSR, Moskva, 142 p.

Guex, J., Hungerbühler, A., Jenks, J.F., O'Dogherty, L., Atudorei, V.,Taylor, D.G., Bucher, H. & Bartolini, A. 2010. Spathian (Lower Triassic) ammonoids from western USA (Idaho, California, Utah and Nevada). *Mémoires de Géologie (Lausanne),* 49.

Hatleberg, E. and Clark, D.L., 1984. Lower Triassic conodonts and biofacies interpretations: Nepal and Svalbard. *Geologica et Palaeontologica*, 18: 101-125.

Mosher, L.C., 1968. Triassic conodonts from Western North America and Europe and their correlation. *Journal of Paleontology*, 42(4): 895-946.

Mosher, L.C. 1973. Triassic conodonts from British Columbia and the northern Arctic Islands; Geological Survey of Canada, Bulletin 222: 141-193.

Orchard, M.J. 1994. Conodont biochronology around the Early-Middle Triassic boundary: new data from North America, Oman and Timor. Proceedings of the Triassic Symposium, Lausanne, 1992. *Mémoire de Géologie, Université de Lausanne, Helvetia*, 22: 105-114.

Orchard, M.J. 1995. Taxonomy and correlation of Lower Triassic (Spathian) segminate conodonts from Oman and revision of some species of *Neospathodus*. *Journal Paleontology*, 69: 110-122.

Orchard, M.J. 2005. Multielement conodont apparatuses of Triassic Gondolelloidea. *Special Papers in Palaeontology*. 73: 73-101.

Orchard, M.J., Gradinaru. E., and Nicora, A. 2007a. Conodonts from the Olenekian-Anisian boundary interval at Desli Caira, Dobrogea, Romania. *New Mexico Museum of Natural History and Science, Bulletin* 41: 341-346.

Orchard, M.J., Lehrmann, D., Wei Jiayong , Wang Hongmei, and Taylor, H. 2007b. Conodonts from the Olenekian-Anisian boundary beds, Guandao, Guizhou Province, China. *New Mexico Museum of Natural History and Science, Bulletin* 41: 347-354.

Sweet, W.C., 1970. Uppermost Permian and Lower Triassic conodonts of the Salt Range and Trans-Indus Ranges-West Pakistan. In, Stratigraphic boundary problems: Permian and Triassic of West Pakistan, (eds.) Kummel, B. and Teichert, C., *University Of Kansas, Special Publications*, 4: 207-275.
